# Low-temperature H_2_S detection using Fe-doped SnO_2_/rGO nanocomposite sensor

**DOI:** 10.1039/d5ra01664a

**Published:** 2025-07-23

**Authors:** N. B. Thakare, D. N. Bhoyar, U. P. Gawai, V. S. Kalyamwar, K. B. Raulkar, P. S. Bodkhe, G. T. Lamdhade

**Affiliations:** a Department of Physics, Shri Shivaji College of Arts, Commerce and Science Akola 444001 Maharashtra India; b Department of Physics, Shri Shivaji Science and Arts College Chikhli Buldana 444302 Maharashtra India; c Department of Physics, DDSP, Arts Commerce and Science College Erandol Jalgaon 425109 Maharashtra India; d Department of Physics, Bharatiya Mahavidyalaya Amravati 444605 Maharashtra India; e Department of Physics, Vidya Bharti Mahavidyalaya Amravati 444605 Maharashtra India gtlamdhade@rediffmail.com; f Department of Chemistry, Vidya Bharti Mahavidyalaya Amravati 444605 Maharashtra India

## Abstract

A low-temperature H_2_S gas sensor was designed using 3% Fe-doped SnO_2_/rGO nanocomposite as the sensing material. Fe-doped SnO_2_ quantum dots (QDs) were prepared using a sol–gel combustion method, subsequently leading to the formation of the Fe–SnO_2_/rGO nanocomposite through a simple sonication process. To evaluate the performance of the sensor material, the sample underwent comprehensive characterization using XRD, FE-SEM, HRTEM, Raman shift, XPS and BET surface area analysis based on nitrogen (N_2_) adsorption–desorption. The XRD pattern HR-TEM confirmed the formation of a well-defined tetragonal crystal phase of SnO_2_, indicating high structural integrity. Meanwhile, the BET analysis revealed a specific surface area of 72.7 m^2^ g^−1^ with pore size of 7.83 nm. Morphological analysis (HR-TEM) revealed that 3% Fe-doped SnO_2_ QDs was uniformly dispersed on the rGO surface, with an average particle size of 5.6 nm. Gas sensing performance of pristine SnO_2_ (S1), 3% Fe-doped SnO_2_ QDs (S2), and 3% Fe–SnO_2_/rGO (S3) nanocomposite based sensors was evaluated at operating temperatures ranging from 25 °C to 175 °C. Incorporation of rGO significantly enhanced the sensitivity of the 3% Fe-doped SnO_2_/rGO nanocomposite towards H_2_S compared to pristine SnO_2_ and 3% Fe–SnO_2_ QDs. The 3% Fe–SnO_2_/rGO (S3) based sensor demonstrated a significant response of about 42.4 to 10 ppm H_2_S at a low operating temperature of 100 °C, with a rapid response time of 21 seconds. It also exhibited excellent selectivity for H_2_S against interfering gases such as NH_3_, LPG, and CO. The enhanced sensitivity and selectivity are attributed to the synergistic interaction between 3% Fe–SnO_2_ and rGO. A possible gas sensing mechanism underlying the improved performance of the nanocomposite is discussed.

## Abstract 2

Gas sensing mechanism of 3% Fe–SnO_2_/rGO nanocomposite: The sensor exhibited a high response of 42.4 to 10 ppm H_2_S at 100 °C, with excellent selectivity and a rapid response time.

## Graphical abstract

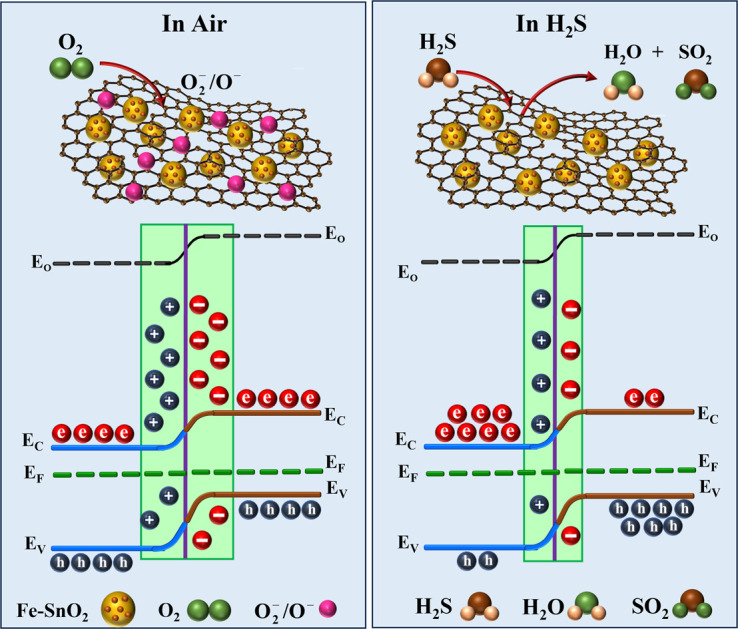

## 1. Introduction

Hydrogen sulfide (H_2_S) is a colorless, flammable, and hazardous gas that acts as both an irritant and an asphyxiant.^1^ It is released from wastewater treatment plants, manure management facilities, pulp and paper mills, swine confinement operations, and the chemical, petroleum, and natural gas industries.^2^ In certain asthmatic patients, prolonged exposure to 2–5 ppm H_2_S might result in bronchial constriction, nausea, headaches, and eye tears.^1,2^ Exposure to 20 ppm can lead to fatigue, reduced appetite, headaches, irritability, memory impairment, and dizziness.^3^ At concentrations of 100 ppm or higher, H_2_S becomes immediately dangerous to life, potentially causing olfactory fatigue or paralysis, pulmonary edema, unconsciousness, coma, and even death.^4^ Communities situated near industrial facilities, landfills, and densely populated regions are especially susceptible to H_2_S exposure. According to the World Health Organization (WHO), under typical working conditions, hydrogen sulfide concentrations in workplace air are generally expected to remain below 7–10 ppm as an 8 hour time-weighted average.^5^ Hence, developing highly selective and sensitive gas sensors for H_2_S detection is essential to safeguard human health and the environment.

Tin oxide (SnO_2_), an n-type semiconductor oxide with a wide band gap of 3.6 eV,^6^ is widely used in gas sensors due to its ability to detect a range of toxic gases and organic vapors, cost-effectiveness, and thermal stability.^7,8^ However, its elevated operating temperature (above 200 °C) and poor selectivity limit its practical applications. Therefore, improvements in sensitivity and selectivity are necessary.^9,10^ These challenges can be addressed by introducing suitable dopants, tuning particle size and morphology, and designing advanced heterostructures.^11–13^

Recently, metal oxide–graphene heterostructures have emerged as attractive candidates for gas sensors because of their enhanced performance at lower temperatures, along with increased sensitivity, selectivity, and rapid response times.^13,14^ Zhilong Songa and colleagues reported a SnO_2_ quantum wire/rGO nanosheet nanocomposite that exhibits the highest sensitivity 8.5 to 50 ppm H_2_S at room temperatures.^15^ Aditya Choudhari *et al.* examined the sensing properties of rGO/SnO_2_ nanocomposites for 100 ppm NO_2_ and noted a peak response of 99.9% at 150 °C.^16^ Niavol *et al.* examined SnO_2_-NPs/rGO nanocomposite with excellent long-term stability and response of 16.77 to 600 ppm at 130 °C.^17^ More recently, Bhangare *et al.* presented a SnO_2_/rGO nanohybrid that demonstrates a response of 3.7 to 2 ppm H_2_S at 200 °C.^18^ The Bi-doped SnO_2_/rGO nanocomposite synthesised Guo *et al.* showed excellent response of 48.6 at 150 °C to 5 ppm benzene.^19^ The Cu–SnO_2_/rGO H_2_S sensor developed by Chen *et al.* displayed ultrahigh sensitivity (*S* ∼ 1415.7) at 120 °C.^20^ It is evident that the performance of SnO_2_-based gas sensors can be significantly enhanced by incorporating graphene and utilizing an appropriate dopant.

In this study, we report 3% Fe–SnO_2_/rGO nanocomposite-based gas sensor for the detection of H_2_S at low operating temperatures. The preference for 3% doping in metal oxide gas sensors stems from its capacity to substantially improve sensing performance while preserving structural and chemical stability. At this concentration, dopant ions effectively tune the electronic structure, enhance the density of surface-active sites, and facilitate the formation of beneficial defects such as oxygen vacancies. Moreover, it minimizes the risk of secondary phase formation, which tends to occur at higher doping levels.^21–24^ The integration of 3% Fe–SnO_2_ quantum dots with rGO significantly enhances gas sensing performance compared to pristine SnO_2_ and 3% Fe–SnO_2_ QDs. The 3% Fe–SnO_2_ QDs provide a large surface area, while the rGO facilitates rapid charge transfer and improved selectivity. The plentiful active sites for gas adsorption, along with the heterojunction interface, facilitate improved charge separation and enhance the gas sensing response. The synergistic effect of rGO and Fe–SnO_2_ QDs enhances the gas sensor's sensitivity, selectivity, response speed, and stability, making it highly efficient for H_2_S detection.

## 2. Experimental details

The analytical reagents listed below were employed without additional purification: tin chloride pentahydrate (SnCl_4_·5H_2_O, Sigma-Aldrich), iron(iii) nitrate nonahydrate (Fe (NO_3_)_3_·9H_2_O, Sigma-Aldrich), urea (CO(NH_2_)_2_, Merck), ammonium hydroxide (NH_4_OH, Merck), and reduced graphene oxide (Ad Nano Technologies Pvt. Ltd, India).

### 2.1 Synthesis of SnO_2_ (S1) and 3% Fe–SnO_2_ (S2)

The sol–gel combustion method was method was employed to produce pristine SnO_2_ nanoparticles (NPs) and Fe-doped SnO_2_ QDs. A uniform solution was obtained by dissolving tin chloride pentahydrate and urea in 200 mL of deionized water in a stoichiometric ratio, followed by stirring at room temperature for 1 hour. After stirring for 60 minutes, ammonium hydroxide (NH_4_OH) was gradually introduced into the solution until the pH reached 7. The mixture was continuously stirred and heated at 80 °C until the precursor solution transformed into a viscous gel. Then, the gel was completely dried by direct heating on the hot plate at 150 °C, resulting in the formation of a black-brown powder. The resultant powder was ground for 30 minutes using an agate mortar and pestle, then calcined in air at 500 °C for 5 hours and cooled naturally to yield crystalline pristine SnO_2_. To synthesize Fe-doped SnO_2_ QDs (Sn_1−*x*_Fe_*x*_O_2_) with 3% Fe doping, a similar procedure was followed, incorporating 0.03 mol of Fe from ferric nitrate nonahydrate (Fe(NO_3_)_3_·9H_2_O) into the precursor solution before gel formation. This ensured homogeneous doping, ultimately yielding 3% Fe-doped SnO_2_ QDs after calcination.

### 2.2 Synthesis of 3% Fe–SnO_2_/rGO (S3)

To synthesize the 3% Fe–SnO_2_/rGO composite, 1 g of 3% Fe–SnO_2_ and 4 mg of rGO were individually dispersed in 200 mL and 40 mL of deionized water, respectively. The solutions were then stirred and sonicated using a 400 W probe sonicator for 15 minutes to ensure uniform dispersion. The dispersed rGO was slowly introduced into the Fe–SnO_2_ suspension while stirring vigorously, followed by an additional 15 minutes of sonication. The obtained mixture was placed in a drying oven at 100 °C for 24 hours. The final product was collected and ground for 30 minutes to obtain a fine powder.

### 2.3 Characterization

A field emission scanning electron microscope (FE-SEM, Carl Zeiss Model Supra 55, Germany) and a high-resolution transmission electron microscope (HR-TEM, JEOL JEM 2100 PLUS) have been employed to examine the morphology of the produced composites. X-ray photoelectron spectroscopy (XPS, Omicron ESCA, Oxford Instruments, Germany) and energy dispersive spectroscopy (EDS) were used to assess the elemental composition. A Cu-Kα radiation source (*λ* = 0.15405 nm) and a Rigaku Miniflex-II (Japan) were used for X-ray diffraction (XRD) studies. Raman spectroscopy (XploRA PLUS, Horiba, Japan), Fourier transform infrared spectroscopy (FT-IR, Affinity-1S IR spectrometer, Shimadzu Corporation, Kyoto, Japan), ultraviolet-visible (UV-Vis) spectroscopy utilizing a UV-Vis NIR spectrophotometer (LAMBDA 750, PerkinElmer), and BET surface area measurements were performed using a Quantachrome NOVA 2200 series volumetric gas adsorption system.

### 2.4 Sensor fabrication

Interdigitated silver electrodes were fabricated using a screen-printing process on ceramic substrates.^21,22^ The ceramic substrates provide a durable, thermally stable, and electrically insulating surface for applying the sensor materials. High-temperature silver conductive paste (Techinstro, India) was screen-printed using a manual screen-printing machine and cured at 120 °C for 10 minutes. The fabricated interdigitated silver electrode consists of 16 digits, each measuring 10 mm in length and 0.4 mm in width, with an interdigit spacing of 0.4 mm between adjacent pairs. Ultrasonically dispersed solutions of the samples (in absolute alcohol) were drop-coated onto the prepared electrodes and allowed to dry in an oven at 100 °C for 24 hours to ensure a smooth surface free of cracks and bubbles before the sensing tests.

### 2.5 Gas sensing measurements

The gas sensing abilities of the synthesized materials were assessed using a gas detection test system, which included a gas chamber, sample holder, Keithley 6487 Picoammeter/voltage source, temperature controller (Nippon NC 2638), and a gas injection system equipped with a mass flow controller (MFC). A steady voltage was applied to the sensor element, and the corresponding current was measured with the Keithley 6487 Picoammeter/voltage source. To create a baseline, the sensing chamber was first flushed with dry air for ten minutes. After that, the analyte gas was introduced to track the sensor response. The sensor was then restored by subjecting it to a dry air flow. The response of the sensor (*S*) is defined as *S* = *I*_g_/*I*_a_, where *I*_g_ and *I*_a_ denote the sensor's electrical current in the presence of the target gas and in air, respectively. Response time refers to the time needed for a gas sensor to achieve 90% of its maximum signal change after exposure to the gas being monitored. Recovery time, on the other hand, is the time taken for the sensor to return to 90% of its baseline value after the target gas is removed.

## 3. Results and discussion

### 3.1 Crystallographic study

**Fig. 1:**
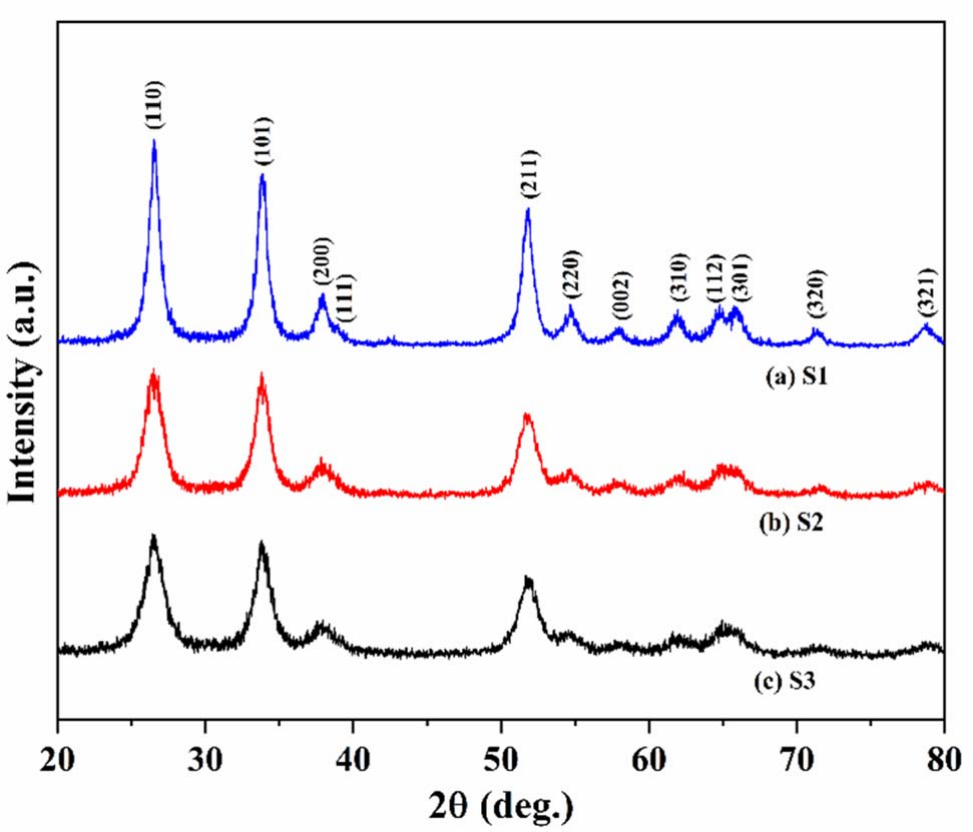
X-ray diffraction pattern of samples (a) S1 (b) S2 and (c) S3 respectively.

Fig. 1 presents the X-ray diffraction patterns obtained for the assessment of phase purity and crystal structure of the S1, S2, and S3 samples, respectively. The XRD peaks identified for sample S1 are closely match the standard JCPDS no. 41-1445, confirming the tetragonal rutile phase of the SnO_2_ nanostructure. The diffraction peaks at 2*θ* values 26.5°, 33.8°, 37.7°, 51.6°, 54.5°, 57.8°, 61.8°, and 65.8° diffraction peaks correlate to the Miller indices (110), (101), (200), (211), (220), (002), (310), and (301), in that order. The full width at half maximum (FWHM) values were determined using Gaussian fitting applied to the diffraction peaks. The Scherrer equation was utilized for calculating the average sizes of the crystallites: *D*= *kλ*/*β* cos *θ*, here *D* represents the crystallite size, *k* is the Scherrer constant (0.94), *β* stands for the FWHM of the diffraction peak, *λ* indicates the incident X-ray wavelength, and *θ* refers to the diffraction angle. The mean crystallite sizes of the particles for samples S1, S2 and S3 are found to be 9 nm, 5.95 nm, and 5.7 nm, respectively. The XRD results are consistent with HR-TEM patterns. The lattice parameters were computed as follows: *a* = *b* = 4.7440(3) Å, *c* = 3.1778(1) Å for SnO_2_; *a* = *b* = 4.7582(12) Å, *c* = 3.1874(5) for 3% Fe–SnO_2_ and *a* = *b* = 4.7582(12) Å, *c* = 3.1874(2) for 3% Fe–SnO_2_/rGO. These values align well with those reported in the literature.^20,25–27^

No further reflections have been detected, thereby ruling out the existence of any additional crystalline phase. The diffraction peaks observed in the S2 and S3 samples are align with the standard tetragonal SnO_2_ phase.^27^ The lack of rGO diffraction peaks in S3 is likely due to the quite low rGO content.^20^ The S3 composite shows a rise in FWHM and a reduction in peak intensity, indicating that Fe^3+^ ions have replaced some of the Sn^4+^ ions in the SnO_2_ lattice, resulting in a smaller crystallite size.^28^ This substitution disturbs the charge balance of the undoped matrix, potentially creating additional oxygen vacancies to maintain charge neutrality due to their low formation energy.^29^

### 3.2 Morphological investigation

**Fig. 2:**
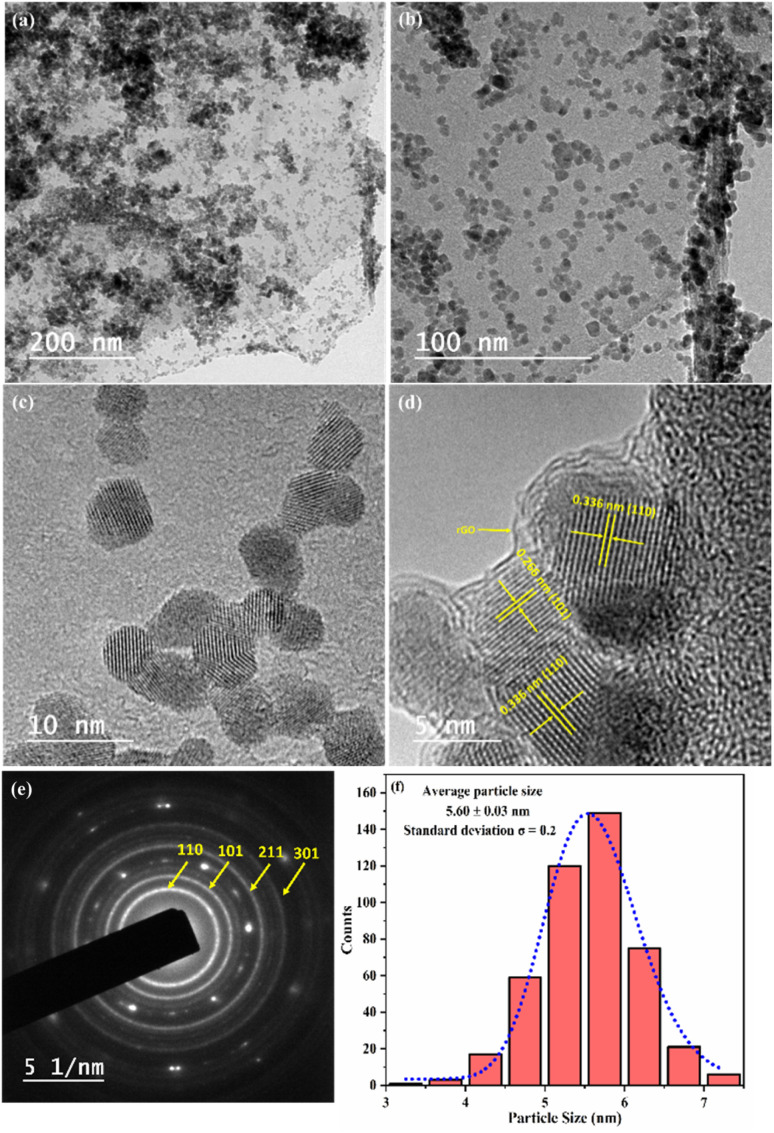
(a–d) Low and high resolution HR-TEM micrographs (e) SEAD pattern and (f) particle size histogram of S3 nanocomposite.

**Fig. 3:**
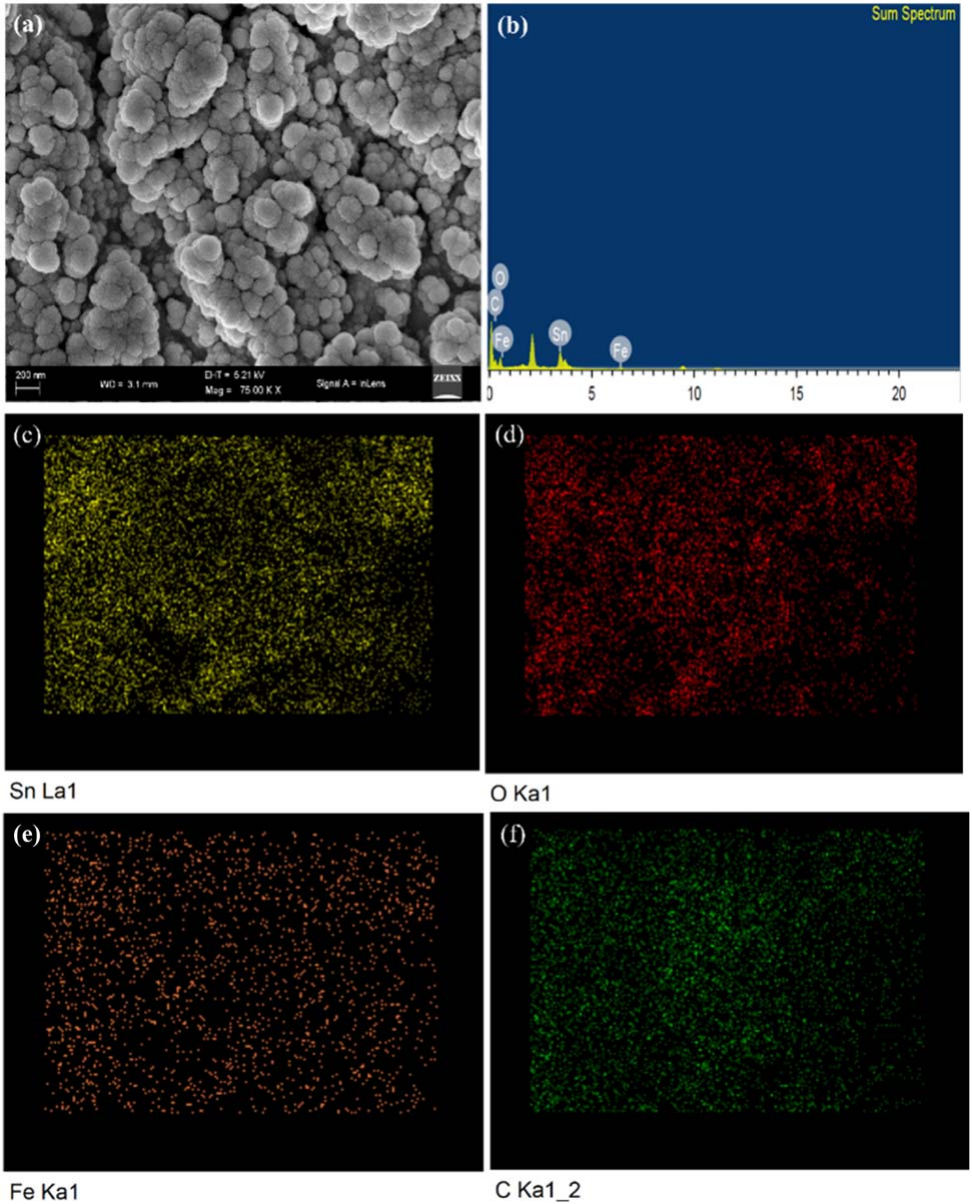
(a) FE-SEM, (b) EDX spectrum and elemental mapping of the (c) Sn (d) O (e) Fe and (f) C in S3 nanocomposite.

**Fig. 4:**
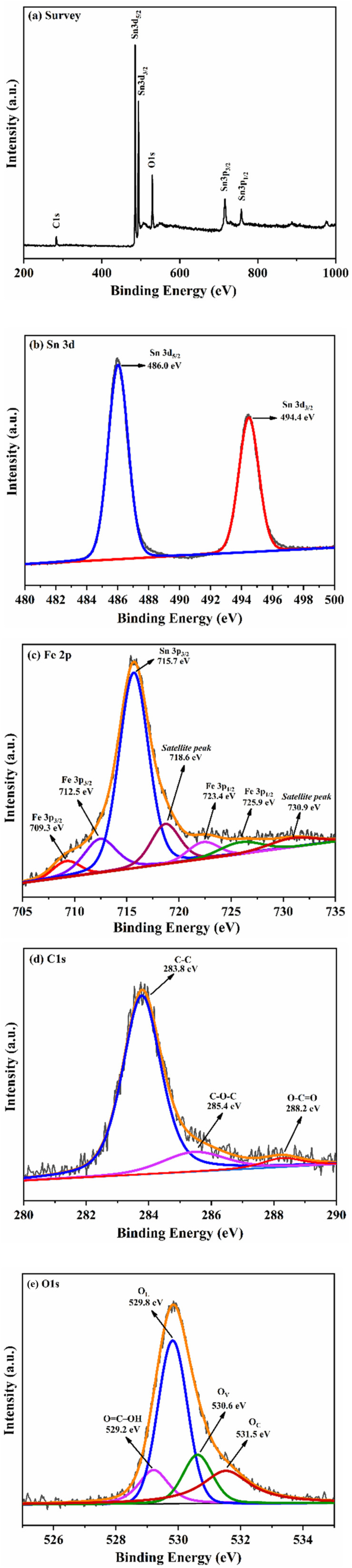
(a) XPS survey spectrum, (b) Sn 3d, (c) Fe 2p, (d) C 1s XPS and (e) O 1s core level XPS spectrum of S3 nanocomposite.

Fig. 2 displayed the HR-TEM micrographs of the S3 nanocomposite. A low-magnification HR-TEM micrograph (Fig. 2(a)–(c)) shows that random-shaped 3% Fe–SnO_2_ QDs are attached to the surface of rGO laminates, with exposed graphene visible at the edges of the material. The HR-TEM micrograph (Fig. 2(d)) shows that the sample has a lattice spacing of 0.336 and 0.266 nm, which corresponds to the (110) and (101) planes of the tin dioxide crystals. The pronounced lattice edges of the 3% Fe–SnO_2_ QDs indicate a significant degree of crystallinity. The four well-defined diffraction rings for the Selected Area Electron Diffraction (SAED) patterns (Fig. 2(e)) corresponded to planes (110), (101), (211) and (301), confirming the tetragonal rutile structure of SnO_2_. The results further substantiate the nonexistence of alternative phases, such as SnO or Fe_2_O_3_, suggesting that Fe ions replace Sn^4+^ ions which is feasible due to the smaller ionic radius of Fe^3+^ (0.063 nm) ions than Sn^4+^ (0.069 nm).^30^ The SAED fringe pattern results align well with the peaks observed in the XRD analysis. Furthermore, the nanoparticle size in S3 nanocomposite (Fig. 2(f)) was determined using Nano Measure software by measuring more than 400 particles. The average particle size, determined from a Gaussian fit of the size distribution histogram, is about 5.60 nm with a standard deviation of *σ* = 0.2 nm. This value shows excellent agreement with the particle size estimated from powder XRD analysis. Fig. 3(a) displays the FE-SEM images of S3 nanocomposite. It was found that S2 QDs might have become agglomerated over rGO laminates due to their high surface energy and the clumping of the smaller particles. The S3 sample matrix provides ample diffusion channels and adsorption sites, which makes gas molecules easier to enter the inner film and more sorption sites can be used. Energy dispersive X-ray spectroscopy (EDS) and elemental mapping of the 3% Fe–SnO_2_/rGO nanocomposite was carried out to ensure the composition and distribution of the elements. The EDS spectrum (Fig. 3(b)) confirms that the samples consist of the elements Sn, O, Fe, and C. The elemental maps (Fig. 4(c–f)) reveal that Sn, O, Fe, and C are uniformly distributed throughout the sample, confirming the successful formation of a well-dispersed S3 nanocomposite.

### 3.3 XPS study

The electronic states and chemical composition of the S3 nanocomposite were examined using XPS spectroscopy. Fig. 4(a) shows the survey spectrum, implies the coexistence of Sn, Fe, O, and C in the sample. Two characteristic peaks in the Sn(3d) core level spectrum (Fig. 4(b)) at 486.0 eV (3d_5/2_) and 494.4 eV (3d_3/2_) with a spin–orbit splitting of 8.4 eV clearly indicates that the Sn is present in Sn^4+^ valence state in diverse chemical surrounding.^17^ The peak positions at 715.7 eV (Sn 3p_3/2_) and 757.5 eV(Sn 3p_1/2_) are attributed to Sn^4+^ in the SnO_2_ lattice.^11^ The core-level XPS Fe 2p spectrum (Fig. 4(c)) shows five different peaks: Fe^3+^: (Fe 2p_3/2_: 712.5 eV, Fe 2p_1/2_: 725.9 eV), Fe^2+^: (Fe 2p_3/2_: 709.3 eV, Fe 2p_1/2_: 723.4 eV) and a satellite peak at 718.6 and 730.9 eV.^31–33^ The identification of Fe^3+^ was verified through Fe 2p XPS peaks observed at 712.5 eV (2p_3/2_) and 725.9 eV (2p_1/2_), with a spin–orbit splitting of 13.4 eV.^34^ In addition, Fe satellite peaks rule out the occurrence of metallic Fe or similar oxides in the SnO_2_ lattice. Therefore, Fe ions with Fe^3+^ and Fe^2+^ oxidation states are effectively integrated into the SnO_2_ lattice.^35^ The absence of Fe metal clusters aligns with the findings from the XRD results. In the deconvoluted XPS spectrum of C 1s (Fig. 4(d)), the binding energies at 283.8, 285.4, and 288.2 eV correspond to the C–C, C–O, and O–C=O bonds in the S3 nanocomposite.^36,37^ The O 1s XPS spectrum, shown in Fig. 4(e), is segmented into three distinct peaks, linked to binding energies at 529.8 eV (O_L_), 530.6 eV (O_V_) and 531.5 eV (O_C_). The most intense peak, observed at 529.8 eV, is associated with lattice oxygen in the SnO_2_ lattice.^38^ The peak seen at 530.6 eV reflects the existence of oxygen vacancies caused by defects on the S3 nanocomposite surface, which may contribute to the availability of sufficient active sites and enable the adsorption of target molecules resulting in gas sensitivity.^39^ The fragile peak at 531.5 eV may be referred to the chemisorbed oxygen (O_2_^−^) on the S3 nanocomposite surface and indicate Sn–O–C bonding.^36,40^ The Sn–O–C bond facilitates a favourable synergistic interaction between rGO and the Fe–SnO_2_ nanoparticles, favourable for gas sensing. The relative contributions of these components O_L_, O_V_ and O_C_ were about 46.38% for O_L_, indicating the presence of oxygen atoms strongly bonded within the SnO_2_ crystal lattice; 16.80% for O_V_, representing oxygen associated with vacancy or defect sites, which play a crucial role in gas sensing activity; and 26.80% for O_C_, attributed to surface-adsorbed oxygen species such as O_2_^−^ or OH^−^, which are essential for surface reactivity and gas molecule interaction. The extra peak at 529.2 eV corresponds to O=C–OH (carboxyl groups) in rGO, suggesting strong interactions between Fe–SnO_2_ and rGO. This further supports the successful formation of the S3 nanocomposite.

### 3.4 Raman study

**Fig. 5:**
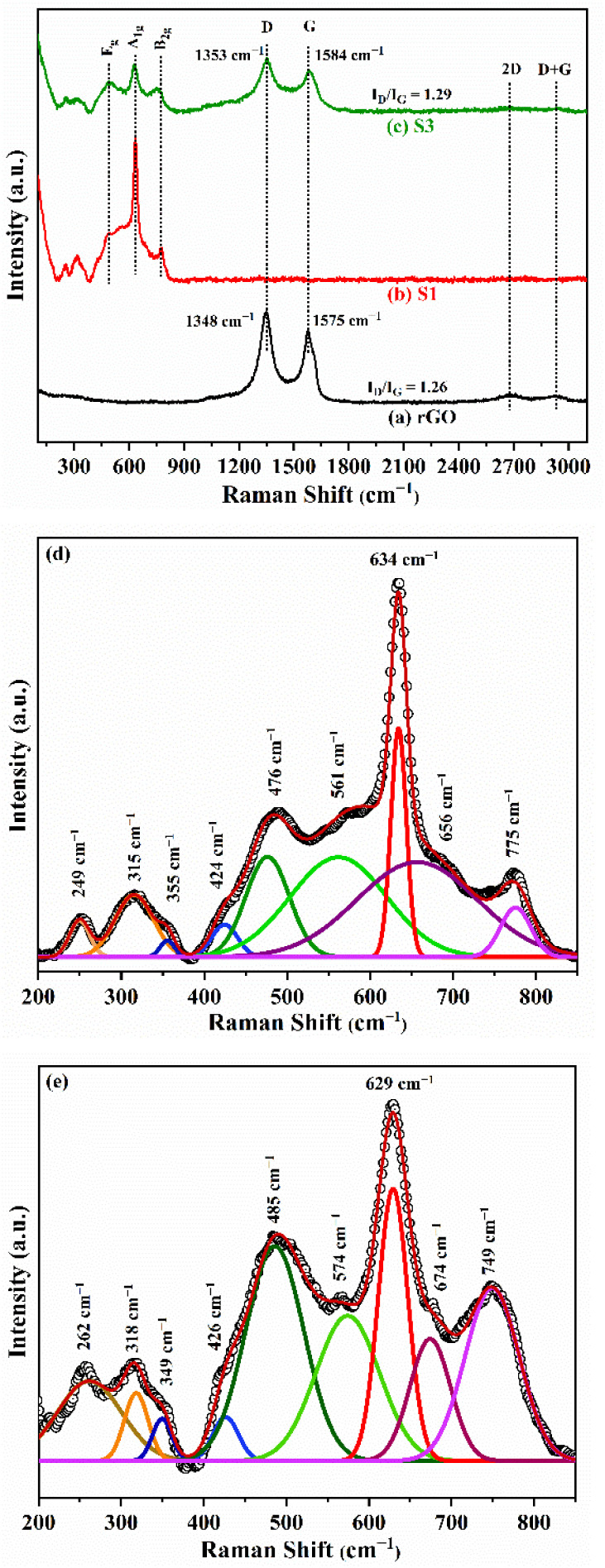
(a) Raman spectra of rGO, (b) S1, and (c) S3, (b and d) deconvoluted Raman spectra of S1 and (c and e) S3 nanocomposite in the wavenumber range 200–850 cm^−1^.

The Raman spectrum of samples rGO, S1 and S3 at room temperature are shown in Fig. 5(a)-(c). The deconvoluted Raman spectrum of S1 in the wavenumber range 200–850 cm^−1^ is shown in Fig. 5(d) and (e). The spectrum of S1 shows three of the four basic active Raman modes: E_g_ (476 cm^−1^), A_1g_ (634 cm^−1^), and B_2g_ (775 cm^−1^). These modes validate the formation of the tetragonal rutile structure of SnO_2_.^41^ In the S3 nanocomposite, the Raman modes: A_1g_ (629 cm^−1^) and B_2g_ (749 cm^−1^) shift to the lower wavenumber range (red shifted), while E_g_ (485 cm^−1^) moves to higher wavenumber (blue shifted). It is clear that the intensity of the characteristic A_1g_ mode decreases, accompanied by peak broadening, due to the substitution of Fe into SnO_2_ in the S3 nanocomposite. The broadening of the A_1g_ mode and the reduction in its intensity suggest a decrease in crystallite size, consistent with the findings from the XRD analysis.^42^ Besides these typical peaks in S1, the other infrared (IR) active and forbidden Raman peaks at 249 cm^−1^, 315 cm^−1^, 355 cm^−1^, 424 cm^−1^, and 656 cm^−1^ correspond to Eu (2) TO, Eu (3) TO, Eu (2) LO, A_2g_, and Eu (2) LO.^6,43^ These modes are visible in the S3 composite at 262 cm^−1^, 318 cm^−1^, 349 cm^−1^, 426 cm^−1^, 574 cm^−1^, and 674 cm^−1^. The peaks observed at 561 cm^−1^ in the S1 and 675 cm^−1^ in the S3 nanocomposite correspond to surface modes. The presence of IR-active/forbidden mode indicates the existence of defects and oxygen vacancies. It is recognized that in an infinite perfect crystal, the scattering of incident radiation is influenced by phonons (with a zero-wave vector *k* ≈ 0) close the zone centre of the Brillouin zone.^44^ As stated by Abello *et al.*, the *k* ≈ 0 selection rule becomes less restrictive when the particle size decreases to the nanoscale, as oxygen vacancies formed during synthesis disrupt lattice periodicity and introduce surface defects.^45^ As a result, both phonons with a zero wave vector (*k* ≈ 0) and those with *k* > 0 participate in Raman scattering, causing a shift and broadening of the Raman mode. The relaxation of the *k* = 0 selection rule induced by the bridging oxygen vacancies allows IR-active Raman modes to become Raman active in the wavenumber range 240–360 cm^−1^.^46^ The Raman mode A_1g_ reflects the vibration of oxygen atoms around Sn ions into the normal plane on the *c*-axis [001] and responds strongly to fluctuations of the O ions.^43^ The red shift of the A_1g_ mode strongly depends on existence of bridging oxygen vacancies (O_V_) on SnO_2_ surface.^47^ Thus, in this study, the shift in the position of the A_1g_ mode from 634 cm^−1^ to 629 cm^−1^ is ascribed to a higher concentration of bridging oxygen vacancies in the S3 nanocomposite. The B_2g_ mode at 775 cm^−1^ in S1 undergoes a red shift and eventually converges to the position of the Eu (LO) mode at 749 cm^−1^ in the S3 nanocomposite. The red shift and the decrease in peak intensity led to the emergence of a broad peak, indicate the presence of a certain concentration of bridging oxygen vacancies (O_V_).^48^ In the S3 nanocomposite, the E_g_ Raman mode exhibits broadening and a shift to a higher wavenumber (485 cm^−1^). This blue shift is attributed to strong phonon confinement caused by oxygen vacancies.^49^ The A_2g_ mode, which features the vibration of Sn and oxygen atoms in the *c*-axis direction, is usually inactive in infrared (IR) measurements.^50^ This mode appears at 424 cm^−1^ in S1 and at 426 cm^−1^ in S3 nanocomposites. Its occurrence is permitted due to reduced symmetry and may be linked to the presence of deeper oxygen vacancies (O_V_).^51^ Additionally, the Raman scattering mode related to O_V_s appears at 561 cm^−1^ and 574 cm^−1^ in both pristine S1 and S3 nanocomposites.^52,53^ In the Raman spectrum of rGO Fig. 5(a), the D mode arising from disordered carbon and the G mode arising from disordered sp^2^ hybridized carbon are at 1348 cm^−1^ and 1575 cm^−1^, respectively.^54^ The low-intensity 2D peak around 2676 cm^−1^ suggests that the reduced graphene oxide is composed of a few layers.^55^ Other band D + D′ around 2920 cm^−1^ indicate presence of ample defects.^56^ The S3 nanocomposite (Fig. 5(c)) exhibits the presence of D and G modes at 1353 cm^−1^ and 1584 cm^−1^ confirms the successful formation of composite. The slight blue shift (9 cm^−1^) of the G band in the S3 composite relative to rGO results from the interaction between Fe–SnO_2_ nanoparticles and rGO within the composite.^57^ The D-to-G peak intensity ratio (*I*_D_/*I*_G_) in S2 nanocomposite (1.29) slightly higher that of rGO (1.26), indicating an increase in defects and disorders (including vacancies and grain boundaries) due to a decrease in the size of in-plane sp^2^ domains.^16^ This can be ascribed to the attachment of Fe–SnO_2_ nanocrystals onto the surface of rGO sheets or their insertion between the rGO layers, which disturb the structural integrity of the rGO sheets.^17,58^

### 3.5 UV-visible absorbance spectra

**Fig. 6:**
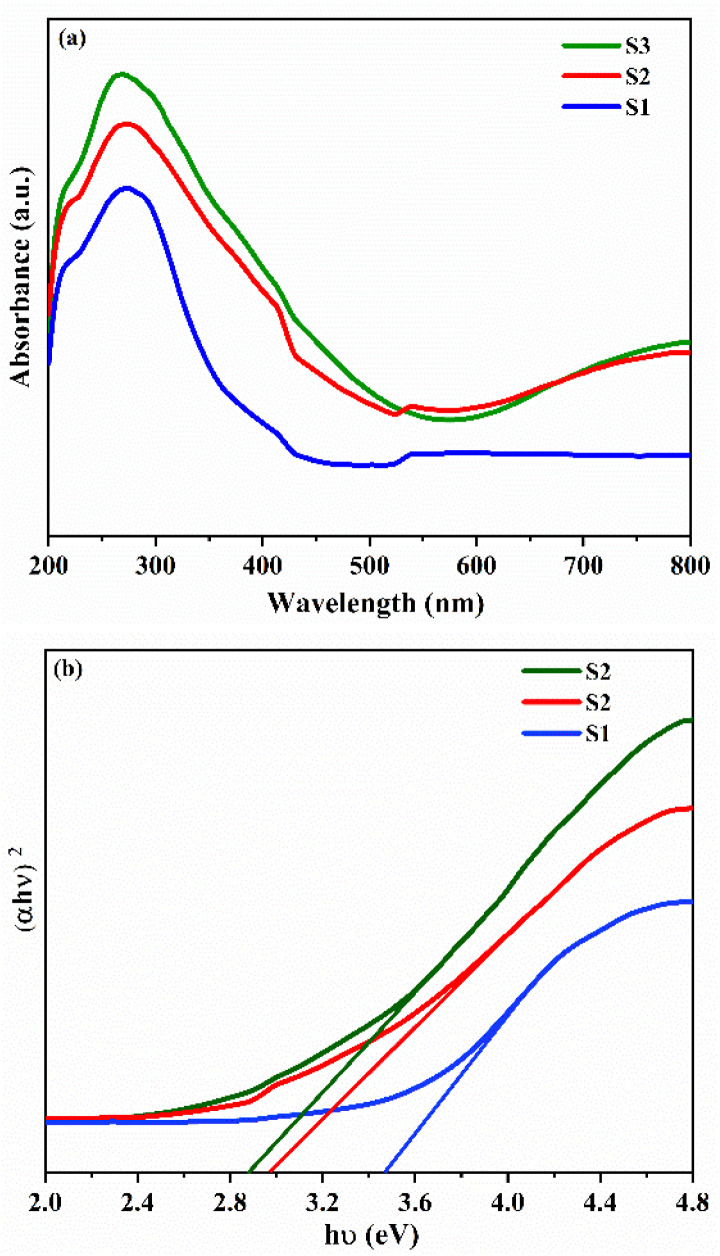
(a) UV-Vis absorption spectra and (b) Tauc plot of S1, S2 and S3 respectively.

Fig. 6(a) presents the UV-Vis absorption spectra of the prepared samples over the spectral range of 200–800 nm. All prepared samples exhibit absorption peaks in the UV region, spanning the range of 264 to 376 nm. In the visible light region, the S3 nanocomposite exhibits greater light absorption efficiency than the S1 and S2 samples, with a steady rise in absorption intensity observed throughout the spectrum. Fig. 6(b) shows Tauc plot to determine the band gap of samples. Energy band gap was estimated using the Tauc equation: *α*ℏ*ν* = *A*(ℏ*ν* − *E*_g_)^*n*^, where ℏ represents Planck's constant, *ν* is the photon frequency, *E*_g_ denotes the band gap, *A* is a constant, *α* stands for absorption, and *n* is a factor based on the type of electron transition. For direct allowed transition *n* = 1/2 and for indirect allowed transition *n* = 2.^59^ The calculated energy bandgap of S1: 3.47 eV, which is smaller than that of bulk SnO_2_ (3.6 eV), while for S2 and S3 it is 2.97 and 2.89 eV, showing a trend of narrowing. The bandgap value typically increases relative to the bulk material when the particle size approaches the Bohr exciton radius (about 2.4 nm for SnO_2_) as a result of quantum confinement effect.^60^

In the present case, a decrease in the bandgap may be ascribed to defects or oxygen vacancies resulting from the inclusion of Fe into the SnO_2_ matrix.^19^ The narrower bandgap indicates that it is much easier for electrons to transfer from the valence band to the conduction band and to cross the p–n heterojunction, which can enhance the gas sensitivity.^20^ Furthermore, the decreased barrier for electron transition contributes to lowering the working temperature of the S3 nanocomposite based sensor.^61^

### 3.6 FTIR analysis

**Fig. 7:**
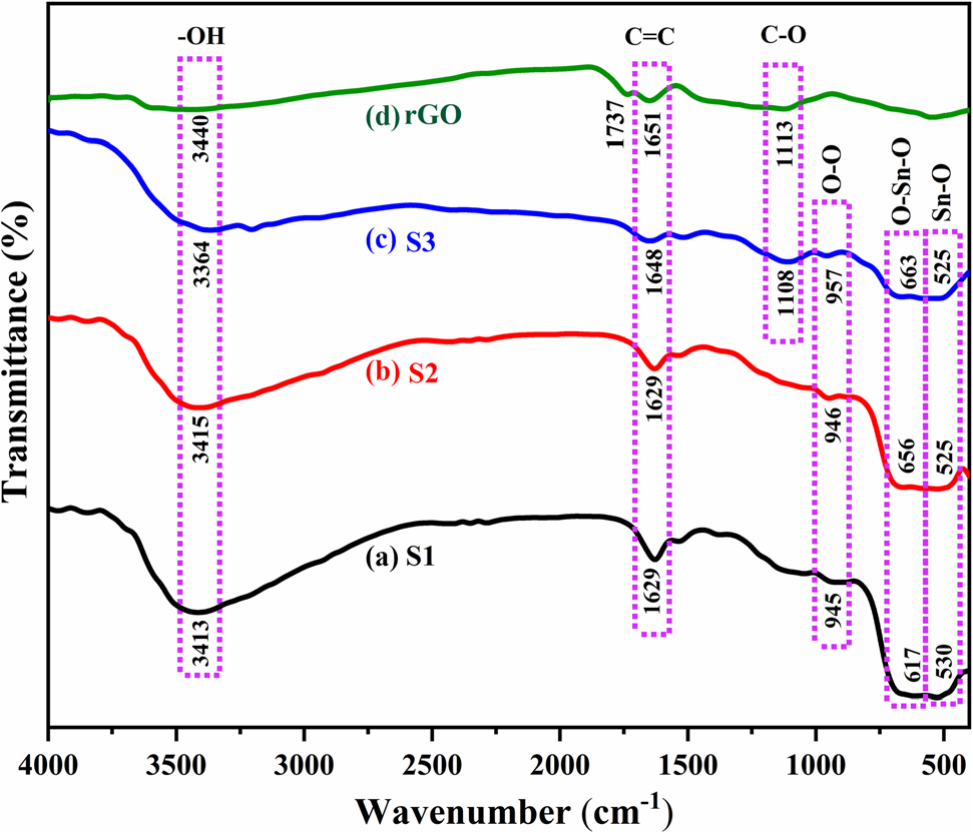
FTIR spectra for (a) S1, (b) S2 (c) S3 and (d) rGO respectively.

The FTIR spectra of rGO, S1, S2, and S3 nanocomposite samples, shown in Fig. 7, reveal the existence of various functional groups within the material. The FTIR spectra of rGO display distinct peaks at 1113, 1651, 1737 and 3440 cm^−1^, indicating the presence of C–O, C=C, C=O and O–H functional groups, respectively.^62^ The FTIR spectra of S1, S2, and S3 exhibit absorption peaks between 3000 and 3700 cm^−1^, which correspond to the O–H stretching vibrations of hydroxyl groups linked to absorbed or adsorbed water.^63^ The absorption peaks at 1629 and 1648 cm^−1^ is attributed to the bending vibration of water molecules, trapped in the samples.^41^ The absorption peaks observed between 500–700 cm^−1^ are assigned to Sn–O–Sn vibrations.^28,61^ These findings confirm the confirm the presence of SnO_2_ crystalline phase in S1, S2 and S3 samples. Additionally, the stretching vibration of O–O resulting from oxygen adsorption on the SnO_2_ surface leads to the appearance of an absorption peak in all these samples at around 950 cm^−1^.^10^ The FTIR spectrum of the S3 nanocomposite shows absorption peaks at 1108 and 1648 cm^−1^, which are closely associated with the C–O and C=C bonds observed in the rGO spectrum. These findings indicate that 3% Fe–SnO_2_ QDs are distributed on the rGO surface. The incorporation of Fe into SnO_2_ resulted in a slight wavenumber shift toward the infrared region, confirming the presence of Fe in the S3 nanocomposite.^64^ The disappearance of the C=O band at 1737 cm^−1^ of rGO indicates that rGO undergoes further reduction due to the attachment of 3% Fe–SnO_2_ QD to rGO during the synthesis process.^65^

### 3.7 Gas sensing performance

**Fig. 8:**
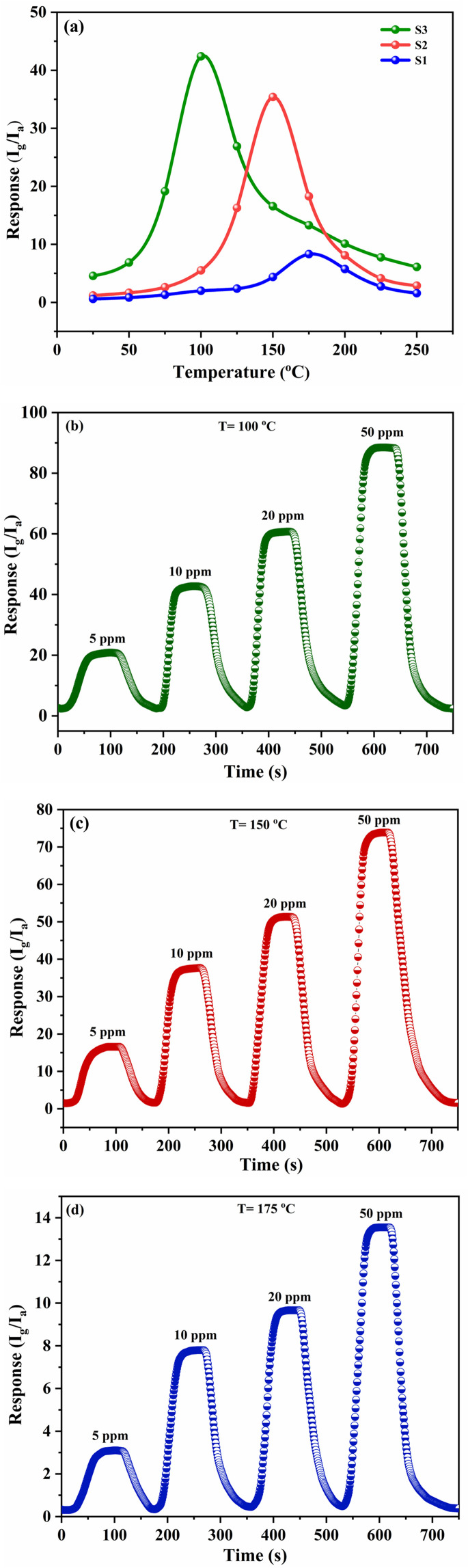
(a) The gas-sensing performance of the S1, S2 and S3 sensors toward 10 ppm H_2_S at various temperatures, (b)–(d) gas sensing curves for H_2_S concentrations ranging from 5 ppm to 50 ppm, for S3, S2 and S1 gas sensors at 100 °C, 150 °C and 175 °C.

**Fig. 9:**
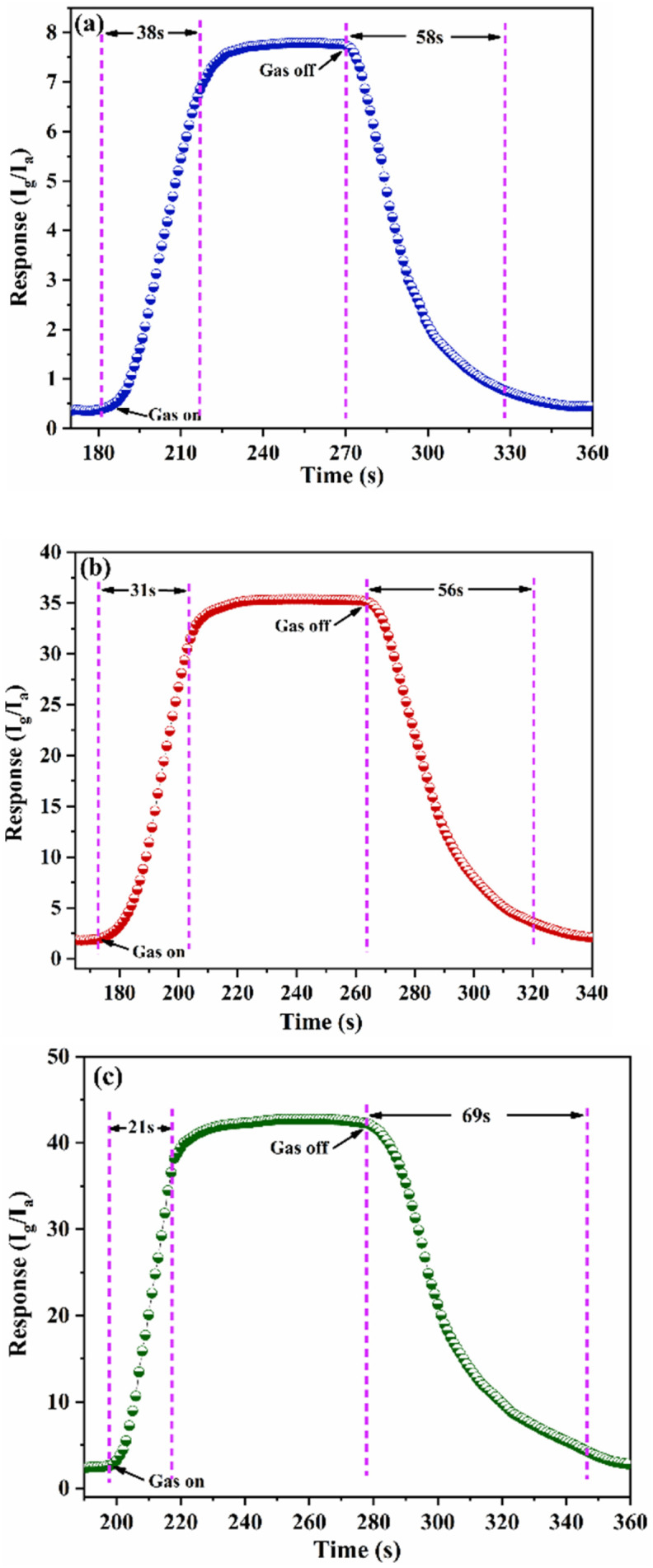
Response recovery time for (a) S1, (b) S2 and (c) S3 to 10 ppm H_2_S.

The gas-sensing performance of the S1, S2 and S3 sensors toward 10 ppm H_2_S at various temperatures are presented in Fig. 8(a). The S3 nanocomposite sensor delivers a peak response of 42.4 to 10 ppm H_2_S at a working temperature of 100 °C. In comparison, the S1 and S2 sensors exhibit responses of 35.3 and 7.8 at operating temperatures of 150 °C and 175 °C, respectively, for the same H_2_S concentration. The Fig. 8(b)–(d) shows the sensing curves for H_2_S concentrations ranging from 5 ppm to 50 ppm, for S3, S2 and S1 gas sensors at 100 °C, 150 °C and 175 °C respectively. It is clear that the sensor's sensitivity rises with an increase in H_2_S gas concentration. The Fig. 9(a)–(c) displays the magnified images for response recovery time of S1, S2, and S3 sensors. For 10 ppm H_2_S, the response and recovery times of the S1-based sensor are measured to be 38 and 58 seconds, respectively, whereas those of the S2-based sensor are 31 and 56 seconds. The S3 nanocomposite-based sensor demonstrates the quickest response time of 21 seconds, with a recovery time of 69 seconds for 20 ppm H_2_S. The S3 nanocomposite sensor functioned efficiently at a low temperature and had the shortest response time (∼21 s) owing to the synergistic effects between Fe–SnO_2_ QDs rGO. The S3 nanocomposite-based H_2_S gas sensor exhibited an extended recovery time, which can be ascribed to multiple contributing factors. Notably, the strong chemisorptive interaction between H_2_S molecules and the SnO_2_ surface, along with the high defect density in the rGO matrix, impedes the desorption process. The the hybrid architecture promotes effective gas adsorption due to its large surface area and abundant oxygen vacancies, these same attributes also delay the release of adsorbed species after gas removal. Furthermore, the sensor's relatively low operating temperature reduces the thermal energy necessary to overcome desorption barriers. As a result, despite its enhanced sensitivity, the sensor demonstrates prolonged recovery behavior.

**Fig. 10:**
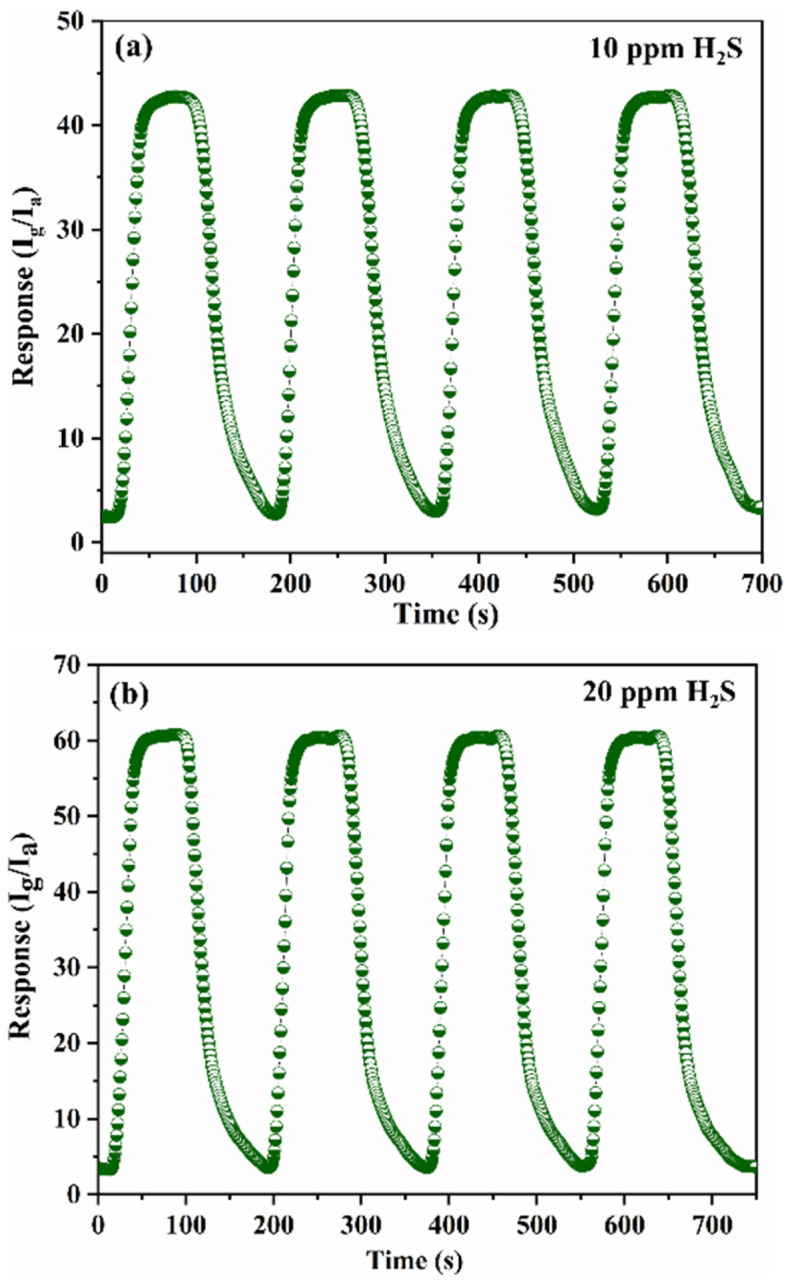
Repeatability of S3 sensor to (a) 10 ppm and (b) 20 ppm H_2_S at 100 °C.

**Fig. 11:**
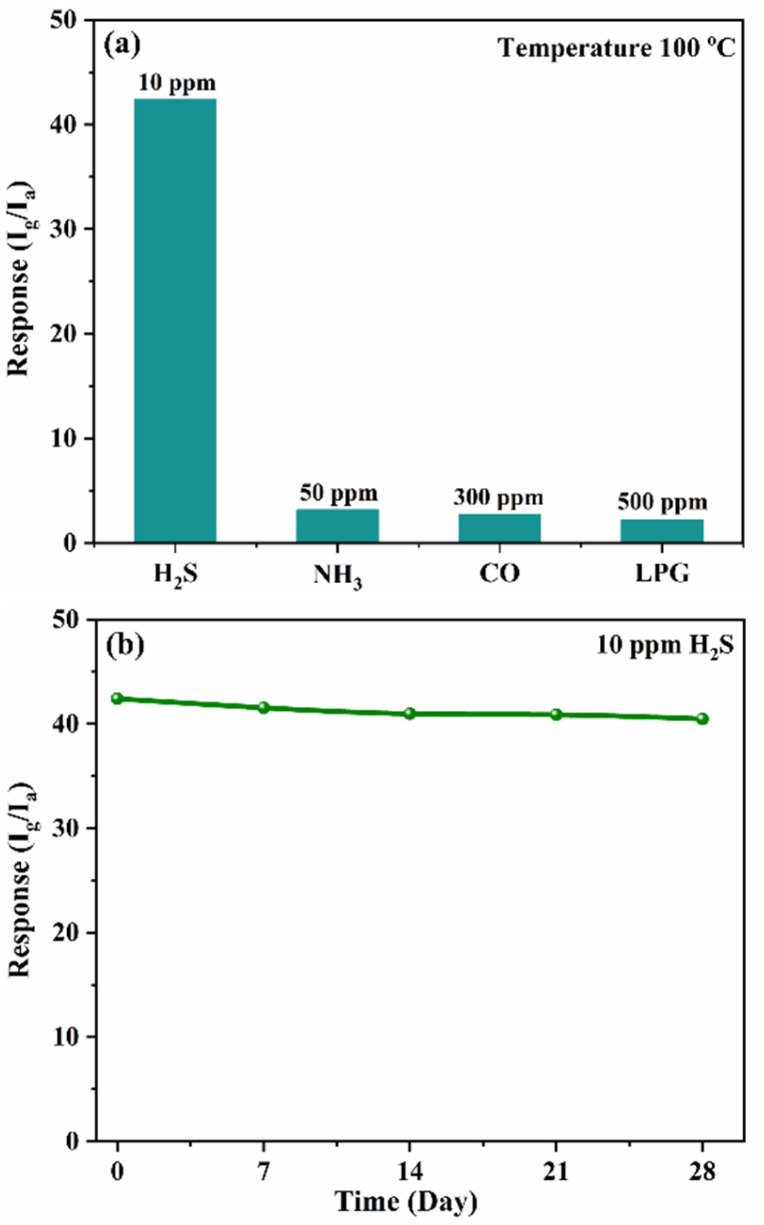
(a) Selectivity of S3 nanocomposite sensor towards NH_3_ (50 ppm), CO_2_ (300) and LPG (500 ppm) at 100 °C, (b) long term stability of S3 nanocomposite sensor.

The sensor exhibits an excellent tracking response with increasing concentrations of H_2_S and demonstrates good recovery in ambient air. Fig. 10(a) and (b) illustrates the repeatability of the S3 sensor upon exposure to 10 ppm and 20 ppm H_2_S. The consistent response across multiple cycles confirms the sensor's excellent repeatability and reliable performance. Fig. 11(a) displays the selectivity of the S3 nanocomposite sensor was tested against NH_3_, CO and LPG. The sensor showed sensitivities of 3.1 for 50 ppm NH_3_ and 2.3 for 300 ppm CO and 2.1 for 500 ppm LPG, highlighting the impressive selectivity for H_2_S compared to NH_3_, CO and LPG. The excellent selectivity for H_2_S might result from its stronger reducing capability, which enables efficient interaction with surface-adsorbed oxygen species at relatively low operating temperatures. Fig. 11(b) presents the long-term stability of the S3 sensor exposed to 10 ppm H_2_S at 100 °C, with measurements conducted at 7 day intervals over 28 days. The minimal fluctuation in sensor response over time indicates stable sensing performance and confirms its long-term operational reliability. The sensor exhibited stable performance with only slight deviation, indicating good long-term stability. However, possible causes of slight performance degradation include surface contamination, strong binding of sulphur species, and prolonged thermal stress, which may induce structural or morphological changes at the heterojunction interface. These effects can reduce the number of active sites and hinder charge transport, ultimately leading to sensor aging. Table 1 summarizes a comparison of the S3 nanocomposite sensor sensing performance with previously reported studies, highlighting that the sensor developed in this work demonstrates superior performance.^21–24,66–71^

**Table 1:** Summary of comparison for the S3 nanocomposite sensor performance towards H_2_S

Material	Concentration (ppm)	Temperature (°C)	Sensitivity	Response time	Recovery time	Ref.
Fe–SnO_2_	100	275	92	NA	NA	21
Cd–SnO_2_	10	275	31	NA	NA	22
Al–SnO_2_	20	350	17.38	35 s	NA	23
Au–SnO_2_	5	370	22	35 s	40 s	24
Fe–SnO_2_	50	250	67.9	<10 s	<15 s	66
SnO_2_	5	125	255	120 s	224 s	67
CuO–SnO_2_	1000	150	84%	53 s	83 s	68
Cu–SnO_2_	100	180	25.3	10 s	42 s	69
Ag–SnO_2_	450	100	1.38	46 s	110 s	70
La–SnO_2_	10	300	96%	20 s	48 s	71
FeSnO_2_/rGO	10	100	42.4	21 s	69 s	Present work

### 3.8 Gas sensing mechanism

The functioning of metal oxide gas sensors is influenced by variations in electrical resistance, driven by temperature-dependent interactions between gas molecules and the sensor surface. Interactions include surface reactions, gas adsorption and desorption. The temperature-dependent oxygen adsorption reaction can be described as follows:

At low temperature (below 100 °C)

1 O_2_(g) + e^−^ ↔ O_2_^−^(ad)

At moderate temperature (100–300 °C)

2 O_2_(g) + 2e^−^ ↔ 2O^−^(ad)

At high temperature (above 300 °C)

3 O_2_(g) + 4e^−^ ↔ 2O^2−^(ad)

**Fig. 12:**
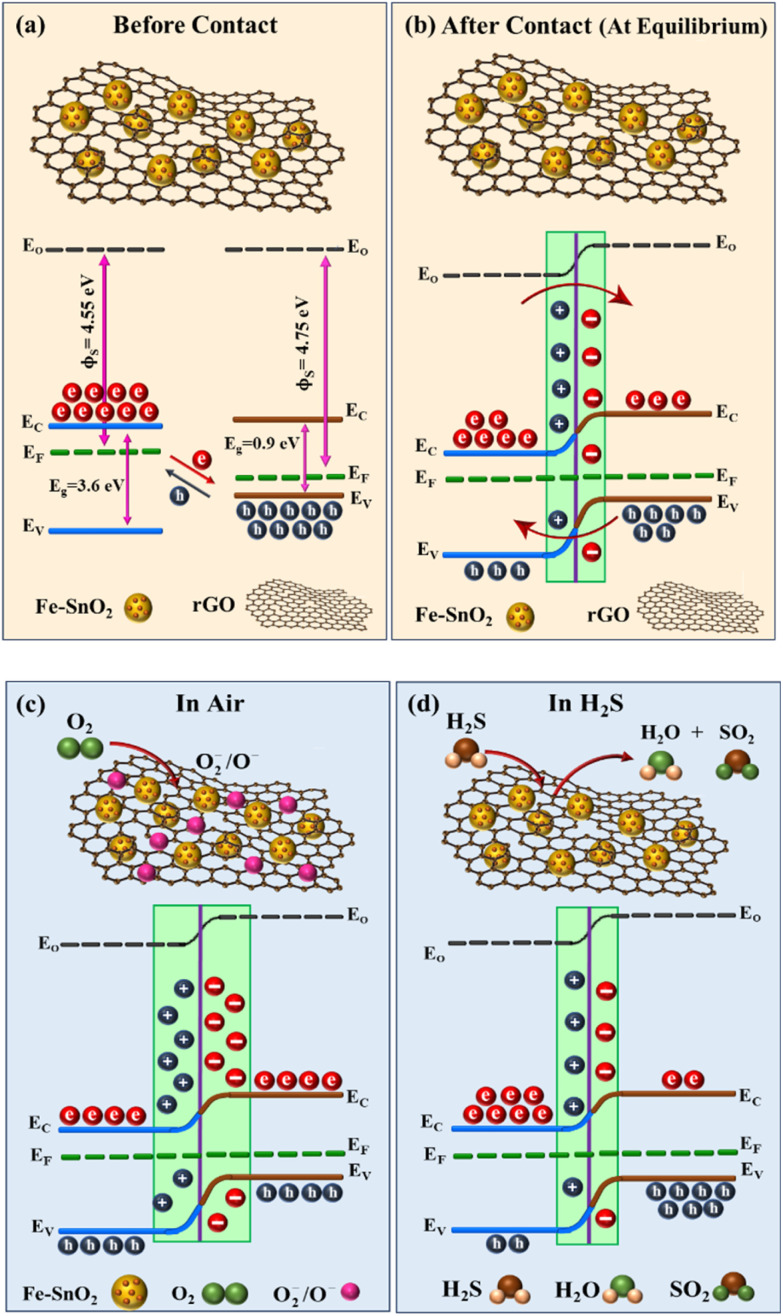
Gas sensing mechanism of S3 nanocomposite sensor in (a) energy band diagram of Fe–SnO_2_ and rGO before contact (b) after contact (at equilibrium) (c) air (d) H_2_S.

The gas sensing mechanism is strongly influenced by the incorporation of Fe into SnO_2_ nanostructures as well as the establishment of p–n heterojunction between Fe–SnO_2_ QDs and rGO. The Fe doping in SnO_2_ nanostructures reduces the bandgap, which in turn reduces activation energy, and adds additional active sites, improving chemisorption and leading to a substantially greater response compared to pristine SnO_2_. The synergistic effect among Fe–SnO_2_ QDs and rGO significantly influences the H_2_S gas sensing capabilities of the S3 nanocomposite at lower temperatures. The proposed gas sensing mechanism of S3 nanocomposite towards H2S is shown in Fig. 12. It is well-established that rGO possesses a higher work function (∼4.7 eV) compared to SnO_2_ (∼4.5 eV), as illustrated in Fig. 12(a), owing to the significant difference in their Fermi level positions.^17,72^ When Fe–SnO_2_ and rGO come into contact, a p–n heterojunction is formed due to the p-type nature of rGO and the n-type semiconducting behavior of SnO_2_.

In the S3 gas sensor, the difference in work functions and carrier concentrations between the n-type SnO_2_ and the p-type rGO drives the diffusion of charge carriers—electrons from SnO_2_ to rGO and holes from rGO to Fe–SnO_2_ across the heterojunction interface. This bidirectional carrier diffusion leads to band bending near the interface, which continues until thermodynamic equilibrium is reached and the Fermi levels align. As a result, a space charge region (depletion layer) forms at the interface, accompanied by the development of a built-in electrostatic field as shown in Fig. 12(b).^73^ This field acts as a potential barrier that influences the charge carrier transport across the junction. When such heterojunction-based sensors are exposed to ambient air at an appropriate operating temperature, oxygen (O_2_) molecules are adsorbed onto the surface of SnO_2_ and the defect sites of rGO. These adsorbed molecules capture electrons from the conduction band of SnO_2_, forming chemisorbed oxygen species such as O_2_^−^, O^−^, depending on the temperature. This electron withdrawal enhances the width of the space charge region at the heterojunction, thereby increasing the sensors resistance in air as shown in Fig. 12(c). Upon exposure to H_2_S, a reducing gas, the pre-adsorbed oxygen species (O_2_^−^, O^−^) on the S3 nanocomposite surface react with H_2_S molecules, resulting in the release of electrons back into the conduction band of SnO_2_ and (as shown in eqn (4)).

4 H_2_S + 3O^−^(ads) → H_2_O(vapor) + SO_2_(g) + 3e^−^

This electron reinjection reduces the width of the electron depletion layer in SnO_2_ and narrows the space charge region at the p–n junction interface as shown in Fig. 12(d). Consequently, the potential barrier between adjacent grains or across the heterojunction is lowered, facilitating carrier transport. This leads to a significant decrease in sensor resistance and a corresponding increase in conductivity, which is characteristic of n-type semiconductor response to reducing gases. Under these conditions, rGO nanosheets support the SnO_2_ framework by offering high-mobility electron pathways, promoting faster interfacial charge transfer and amplifying the sensor's response through synergistic interaction. The removal of the H_2_S gas causes oxygen molecules to re-adsorb on the SnO_2_ surface, which restores the depletion layer. The S3 nanocomposite gas sensor demonstrated superior sensitivity to H_2_S compared to the S1 and S2 sensor. The inclusion of Fe into SnO_2_ reduces the particle size, which enhances the active surface area and creates additional active sites by introducing defects/oxygen vacancies. The catalytic activity of Fe accelerates the reaction kinetics between H_2_S and chemisorbed oxygen. The rGO high surface area, defects and functional groups provide abundant of adsorption sites for target gas. The heterojunction formed at the interface of Fe–SnO_2_ and rGO generates an internal electric field, which facilitates charge carrier separation and enhances sensor performance.^74^ The 2D rGO with a near-zero bandgap provides a highly conductive network that enables rapid electron transfer and can lead to a significant variation in electrical conductivity with a slight change in carrier concentration.^75^ Raman and XPS analysis confirm the existence of oxygen vacancies, which serve as active sites for adsorption and reaction, thereby enhancing the density of adsorbed oxygen species. This not only increases the material's sensitivity to H_2_S, but also improves reaction/recovery time and selectivity.^76^ According to previously reported literature, when the crystallite size (*D*) is reduced to a value comparable to or smaller than 2*L*_*D*_ (for SnO_2_, *L*_*D*_ ∼ 3 nm), a substantial portion of the material becomes involved in surface interactions, leading to a significant change in the material's gas sensitivity.^77^ In the present work, the average particle size of the S2 QDs in the S3 nanocomposite is about 5.6 nm, which is obviously smaller than the 2*L*_*D*_ for SnO_2_ crystallite. Therefore, the S3 nanocomposite sensor exhibits significantly enhanced gas sensing properties for H_2_S at low temperatures.

### 3.9 BET analysis

**Fig. 13:**
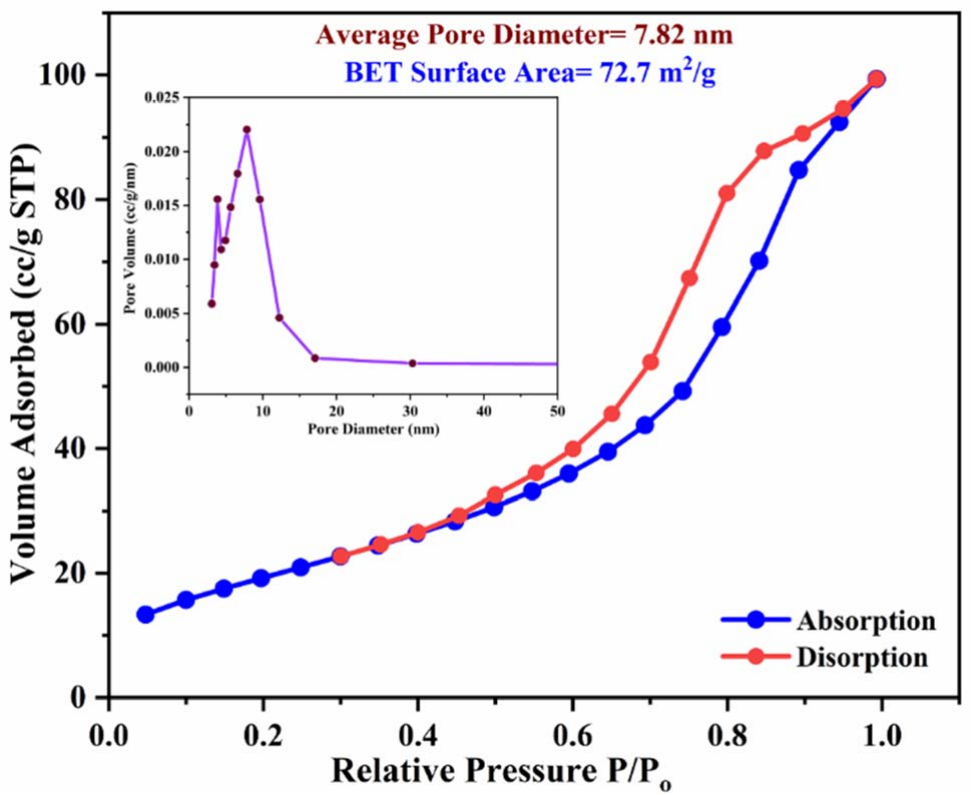
Nitrogen adsorption–desorption isotherm and inset shows BJH pore size distribution plots of S3 nanocomposite.

Fig. 13 depicts the N_2_ adsorption and desorption isotherm, along with the corresponding BJH pore size distribution plot (inset) for the S3 nanocomposite. The adsorption isotherm exhibits a type IV pattern with a hysteresis loop, indicate existence of mesopores (pores 2–50 nm diameter) in the relative pressure range of 0.45 to 1.0, as classified by IUPAC.^78^ The BJH method analysis revealed that the nanocomposite had an average pore size of 7.83 nm, with a pore volume of 0.16 cm^3^ g^−1^. The multi-point BET surface area of the nanocomposite was determined to be 72.7 m^2^ g^−1^. The higher surface area and mesoporous structure provide additional sites for O_2_ adsorption, potentially improving the gas detection abilities of the S3 nanocomposite.

## 4. Conclusions

In conclusion, S2 QDs were successfully synthesized using a sol–gel combustion method, followed by the production of the S3 nanocomposite through a simple sonication process for the fabrication of an H_2_S gas sensor. The S3 nanocomposite exhibited an impressive sensor response (*S* ≈ 42.4) at a lower working temperature of 100 °C, achieving superior performance than samples S2 (*S* ≈ 35.3) and S1 (*S* ≈ 7.8) at operating temperatures of 150 °C and 175 °C, respectively, when exposed to 10 ppm H_2_S. The S3 nanocomposite sensor demonstrated a quick response time (*t* = 21 s) compared to samples S2 (*t* = 31 s) and S1 (*t* = 38 s) when exposed to 10 ppm H_2_S. Furthermore, the nanocomposite sensor demonstrated remarkable selectivity for H_2_S, even when exposed to typical interfering gases like NH_3_, CO, and LPG. The enhanced sensing performance of the S3 nanocomposite is ascribed to the synergistic effects of the p–n heterojunction formed at the interface between 3% Fe–SnO_2_ and rGO. The Fe doping leads to a reduction in the bandgap, which in turn decreases the activation energy required for charge transfer and gas-sensing reactions. Additionally, this process creates defects and oxygen vacancies that offer favourable sites for H_2_S adsorption. At the same time, rGO facilitates efficient charge transport, offers a large surface area, and provides abundant sites for oxygen ion adsorption, all of which contribute to the superior performance of the sensor.

## Author contributions

All authors contributed equally to this work.

## Conflicts of interest

The author declares no competing interests.

## Data Availability

The datasets analyzed during this study are available from the corresponding author upon reasonable request.
